# Video-assisted thoracoscopy in trauma: Case report and review of literature

**DOI:** 10.4103/0970-2113.80335

**Published:** 2011

**Authors:** Angeline N Radjou, Muthandavan Uthrapathy

**Affiliations:** *Perunthalaivar Kamraj Medical College, Pondicherry, India*; 1 *Indra Gandhi Government General Hospital, Pondicherry, India*

**Keywords:** Intrapleural foreign body, trauma, Video Assisted Thoracoscopy

## Abstract

Video Assisted Thoracoscopy (VATS) like any other minimal access surgery has the obvious advantage of less surgical insult, enabling quicker recovery with its attendant reduced hospital costs and earlier return to work. The benefit of earlier return to work is much more important in developing countries. Unfortunately minimal access surgery is yet to gain popularity in trauma surgery, especially in the developing countries due to various reasons. We describe one case report where VATS was used successfully to remove an intrapleural foreign body.

## INTRODUCTION

Minimally invasive surgery has achieved an indispensable role in certain areas of elective general surgery in the last 30 years, even to the extent of being considered the gold standard, e.g., in cholecystectomy. The reduction in surgical insult has led to faster recovery with its attendant advantages. However, minimally invasive techniques have been less favorably adopted by the trauma surgeons the world over, and is yet to gain ground in the developing world which bears 80% of worldwide trauma incidence.

## CASE REPORT

**Figure 1:**
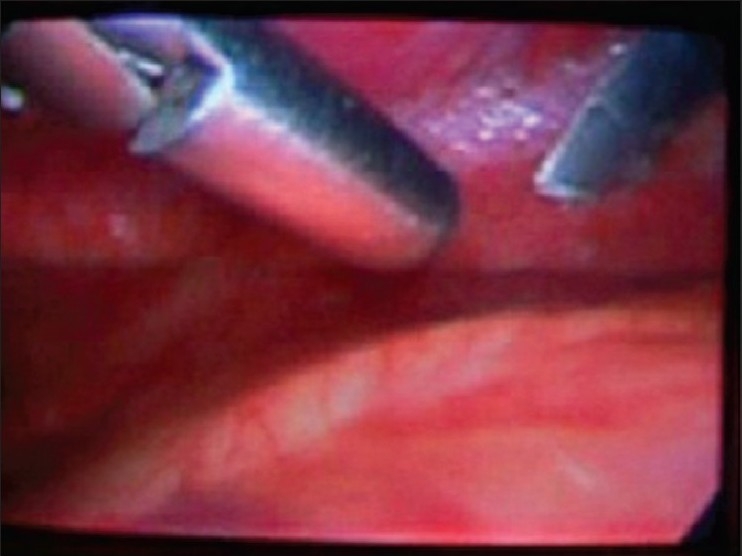
Foreign body on lung surface (L) with endoscopy forceps (R)

**Figure 2:**
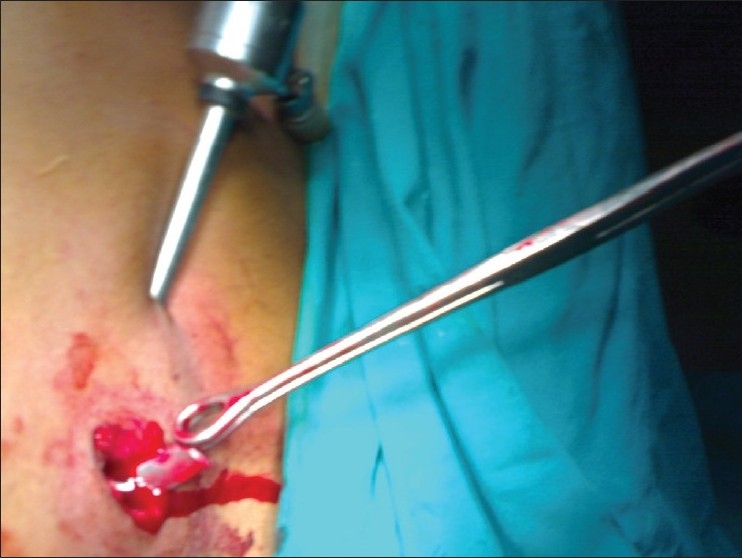
Foreign body being retrieved through main port, at the chest wall

**Figure 3:**
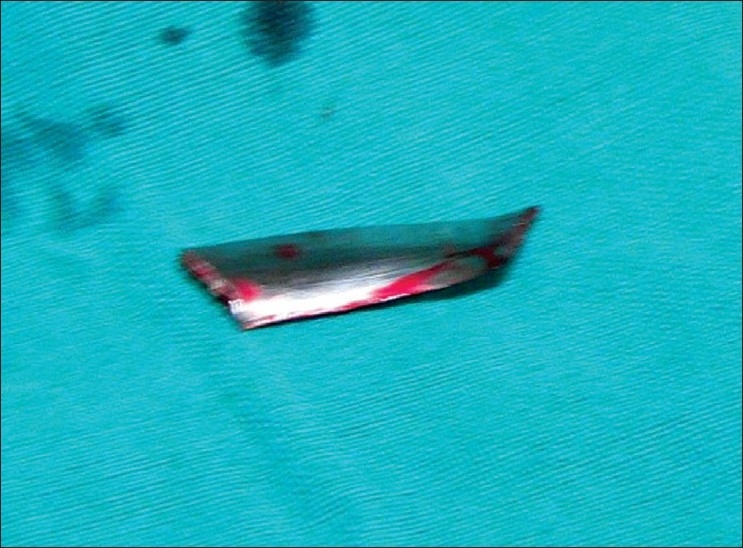
Sharp foreign body retrieved

A 22 year old male patient had sustained chest injury by a metal splinter under moderate velocity. He presented with an entry wound in the 9th intercostal space, anterior axillary line on the right side. He was hemodynamically stable and had no respiratory symptoms or signs and abdomen was normal. Two coordinates of X-ray chest revealed the presence of foreign body in the (R) hemithorax, we suspected that the foreign body could be in the thoracoabdominal cavity based on the mechanism of injury. CT scan confirmed our suspicion that a sharp foreign body was indeed in the (R) thoracic cavity, not close to major vessels. There was a minimal pneumothorax which was not evident clinically. This sharp foreign body mandated removal. We decided to try minimal access surgery but with full preparations for open thoracotomy in case of failure. He needed one lung ventilation as a prerequisite for thoracoscopy. Hence the procedure was done under general anesthesia and intubated with a double lumen tube. He was placed on the anterolateral right thoracotomy position. Single lung ventilation was instituted. In addition CO_2_ insufflation was given up to 5mm Hg, for safer initial trocar placement and better visualization of hemithorax. A 10mm camera port was placed in the (R) 5th intercostal space in the anterior axillary line. A 5 mm working port was placed in the same intercostal space. The foreign body was seen on the inferior surface of the lung Figure 1 and removed through the 10 mm port Figures 2 and 3. The rest of the thoracic cavity and diaphragm were found to be normal. An intercostal tube was placed in the 10 mm port site. He had no adverse event intraoperatively and promptly recovered from anesthesia, with a well expanded lung that was confirmed clinically and with a bedside X ray. However, we still continued to ventilate him electively for 18 hours as our experience with one lung ventilation is limited. He was extubated and ICD removed the same day. He was discharged on the second postoperative day. He is followed up regularly and is doing well.

## DISCUSSION

Thoracoscopy in trauma patients was first described way back in 1976 by Jackson and Ferreira in the diagnosis of diaphragmatic injuries.[1] In the early eighties, Jones *et al*. performed emergency thoracoscopy under local anesthesia for persistent hemothorax thus avoiding thoracotomy.[2]

Even though thoracoscopy and video-assisted thoracoscopy (VATS) are usually used interchangeably there are differences.

Thoracoscopy is a direct three dimensional visualization of the pleura through a single incision.

VATS is a two dimensional view, with multiple ports enabling complex surgical procedures.

Even though thoracoscopy and video assisted thoracoscopy is being used routinely in elective thoracic surgery, it is yet to be more readily adopted in trauma.[3]

VATS scores over thoracotomy with advantages, like better postoperative pain control leading to better lung expansion, shorter time to resumption of normal activity and shorter chest tube duration time.[4,5]

Specific indications of VATS in thoracic trauma include management of retained hemothorax and persistent pneumothorax, evaluation of diaphragm in penetrating thoracoabdominal injuries, evacuation of post traumatic empyema and control of ongoing bleeding in hemodynamically stable patients and rarely for retrieval of foreign bodies, traumatic chylothorax and rib resections.

VATS for trauma can be divided into: Emergency, early and late, depending on the time of performance. Emergency cases are performed on arrival up to trauma day two, mainly for diagnostic purposes, evacuation of retained hemothorax, control of bleeding from intercostal arteries or removal of foreign bodies from the pleural cavity. Early cases are performed from trauma day two to day seven for repair of bronchial injuries and ligation of injured thoracic duct. Late cases are performed after trauma day seven for retained hemothorax, empyema, pleural effusion or pericardial effusion.

Rate of conversion to thoracotomy is only 4% in dedicated and high volume trauma centers. With available evidence VATS is safe and more likely to be successful in the early and late setting.[3] Regarding retrieval of foreign bodies, literature is sparse and mostly only single case reports have been published;[6,7] the largest being a series of four cases in 3 years.[8] Most cases were for the removal of sponges, other iatrogenic material and for the removal of various sharp objects. The advantages with VATS is direct videoscopic visualization of the thorax and mediastinum to remove the sharp object, then to assess and repair the damage at the same sitting in the controlled environment of the operating room is.[9–11] The main contraindication to VATS in trauma patients is hemodynamic instability where an open surgery would be prudent.[12] Prompt recognition of the need to convert to open surgery is of utmost importance.

Even though thoracoscopy had been initiated in trauma work two decades ago, it is yet to pick up momentum by the trauma surgeon. The barriers to the widespread use of VATS in trauma are lack of training of the trauma surgeons in minimal access surgery, large volume of trauma patients in trauma centers, nonavailability of thoracoscopic equipments due to financial constraints or departmental policies.[3] Developing countires are burdened with 80% of worldwide incidence of trauma morbidity and mortality. The bulk of acute trauma careis ultimately borne by government setup in our country which most of the time is under resourced for the overload. With the inevitable improvement in technology and clearer indications for VATS, trauma surgeons should be able to include VATS into protocols for thoracic trauma.[13]

In conclusion, as of date, any minimally invasive surgery has played a minimal role in acute trauma care. The vicious stumbling block is the emergent nature of most trauma surgery and the usual accompanying hemodynamic instability, which is the rule rather than exception in the initial phase of trauma care. This is compounded manifold in developing countries due to the high incidence of trauma, the rudimentary prehospital services and lack of dedicated trauma services, not to mention lack of minimal access surgery skills in trauma surgeon. This results in large volume of seriously injured patients who are invariably unstable hence precluding minimal invasive surgery in acute trauma care. As trauma surgeons we are not able to offer minimal access surgery to the subset of patients who are stable due to lack of skills. This calls for emphasis on minimal invasive surgical training skills to trauma surgeons at least initially in elective general surgery, later to extrapolate to trauma.

## References

[1] Jackson AM, Ferreira AA (1976). Thoracoscopy as an aid to the diagnosis of diaphragmatic injury in penetrating wounds of the left lower chest: A preliminary report. Injury.

[2] Jones JW, Kitahama A, Webb WR, McSwain N (1981). Emergency thoracoscopy: A logical approach to chest trauma management. J Trauma.

[3] Milanchi S, Makey I, McKenna R, Margulies DR (2009). Video-assisted thoracoscopic surgery in the management of penetrating and blunt thoracic trauma. J Minim Access Surg.

[4] Whitson BA, Andrade RS, Boettcher A, Bardales R, Kratzke RA, Dahlberg PS (2007). Video-assisted thoracoscopic surgery is more favorable than thoracotomy for resection of clinical stage I non-small cell lung cancer. Ann Thorac Surg.

[5] Ben-Nun A, Orlovsky M, Best LA (2007). Video-assisted thoracoscopic surgery in the treatment of chest trauma: Long-term benefit. Ann Thorac Surg.

[6] Boulanger B, Lahmann B, Ochoa J (2001). Minimally invasive retrieval of a foreign body after penetrating lung injury. Surg Endosc.

[7] Mironenko ON, Koveshnikov AV, Glushchenko RN (2003). Videothoracoscopic removal of the foreign body from the left pleural cavity. Klin Khir.

[8] Dinka T, Kovács O, Kotsis L (2004). Emergency video-assisted thoracoscopic surgery for intra thoracic foreign bodies. Magy Seb.

[9] Williams CG, Haut ER, Ouyang H, Riall TS, Makary M, Efron DT (2006). Video-assisted thoracic surgery removal of foreign bodies after penetrating chest trauma. J Am Coll Surg.

[10] Hanvesakul R, Momin A, Gee MJ, Marrinan MT (2005). A role for video assisted thoracoscopy in stable penetrating chest trauma. Emerg Med J.

[11] Lang-Lazdunski L, Mouroux J, Pons F, Grosdidier G, Martinod E, Elkaïm D (1997). Role of videothoracoscopy in chest trauma. Ann Thorac Surg.

[12] Cetindag IB, Neideen T, Hazelrigg SR (2007). Video-Assisted Thoracic Surgical Applications in Thoracic Trauma. Thorac Surg Clin.

[13] Degiannis E, Bowley DM, Smith MD (2004). Minimally invasive surgery in trauma: technology looking for an application. Injury.

